# TLR-Mediated Cytokine Gene Expression in Chicken Peripheral Blood Mononuclear Cells as a Measure to Characterize Immunobiotics

**DOI:** 10.3390/genes12020195

**Published:** 2021-01-29

**Authors:** Anna Slawinska, Aleksandra Dunislawska, Arkadiusz Plowiec, José Gonçalves, Maria Siwek

**Affiliations:** Department of Animal Biotechnology and Genetics, UTP University of Science and Technology, 85-084 Bydgoszcz, Poland; aleksandra.dunislawska@utp.edu.pl (A.D.); arkadiop@gmail.com (A.P.); jagoscp@hotmail.com (J.G.); siwek@utp.edu.pl (M.S.)

**Keywords:** *Lactococcus lactis*, *Saccharomyces cerevisiae*, Galactooligosaccharides, Th1/Th2 immune responses, pro-inflammatory

## Abstract

Immunobiotics are probiotics that promote intestinal health by modulating immune responses. Immunobiotics are recognized by Toll-like receptors (TLRs) and activate cytokine gene expression. This study aimed to characterize cytokine gene expression in the chicken peripheral blood mononuclear cells (PBMC) stimulated with purified TLR ligands and live probiotics. PBMC were isolated from the whole blood. PBMC were stimulated with: lipopolysaccharide (LPS), CpG ODN, Pam3CSK4, Zymosan, galactooligosaccharides (GOS), *Lactococcus* *lactis* subsp. *cremoris* (*L. lactis*), and *Saccharomyces cerevisiae* at 42.5 °C and 5% CO_2_ for 3 h, 6 h, and 9 h. After each time-point, PBMC were harvested for RNA isolation. Relative gene expression was analyzed with RT-qPCR for cytokine genes (*IL-1β*, *IL-2*, *IL-3*, *IL-4*, *IL-6*, *IL-8*, *IL-10*, *IL-12p40*, and *IFN-ɣ*) and reference genes (*ACTB* and *G6PDH*). Genes were clustered into pro-inflammatory genes, Th1/Th2 genes, and Th1-regulators. The gene expression differed between treatments in *IL1-β*, *IL-6*, *IL-8*, *IL-10*, and *IL-12p40* (*p* < 0.001). The genes *IL-1β*, *IL-6*, and *IL-8* had the highest fold change of mRNA expression at 3 h in response to TLR ligands. *L. lactis* up-regulated the pro-inflammatory genes at the 6 h time-point. *L. lactis* did not activate the anti-inflammatory *IL-10* gene, but activated *IL-12p40* at 6 h. Hereby, *L. lactis* was proven to exert immunostimulatory properties in PBMC.

## 1. Introduction

The immune responses include two major components: innate and adaptive immunity. Innate immunity identifies the general threats, whereas adaptive immunity occurs after exposure to the specific antigen, such as pathogen or vaccination. Lymphatic cells involved in both types of immune responses produce cytokines, which are small soluble proteins that direct the immune responses. All activated lymphatic cells can secrete cytokines, but predominantly cytokines are produced by different classes of Th lymphocytes involved in cell-mediated immunity [1]. The role and structure of the cytokines as well as their genetic makeup has been well characterized in mammals [2]. In chicken, the cytokine repertoire resembles the mammalian one, including the genetic homology of the cytokine genes [3,4,5]. The transcription of the cytokine genes is initiated when the signal from the antigen is recognized by the receptor and transduced to the nucleus of the lymphatic cell.

The most conserved group of antigens is called pathogen-associated molecular patterns (PAMPs) or microbe-associated molecular patterns (MAMPs). There are several types of PAMPs and MAMPs, which are expressed on the surface of certain classes of microorganisms. For example, lipopolysaccharide (LPS) is present on the membrane of Gram-negative bacteria, lipoteichoic acid (LTA) is present on the membrane of Gram-positive bacteria, and flagellin is characteristic for flagellated bacteria [6]. PAMPs and MAMPs are structurally conserved, absent in the host, and important for the microorganisms [7]. These molecular structures are identified by pattern recognition receptors (PRRs) present on the cells of the innate immune system [8]. The best-characterized group of the molecular receptors, which belong to PRRs and translate the information from the environment to the host immune system, is the family of Toll-like receptors (TLRs). The eukaryotic cells are stimulated by the TLRs to produce antimicrobial peptides, cytokines, and chemokines [7]. PAMPs or MAMPs, also known as TLR ligands, promote certain cytokines that influence the differentiation of the naïve Th cells towards Th1 or Th2 phenotype and subsequently various types of immune responses of the host [9]. Each of the TLRs has a particular ligand specificity, for example, TLR4 binds LPS and TLR2 binds LTA [7].

Immunobiotics are particular strains of probiotic bacteria that promote intestinal health by activating antigen-presenting cells and modulating immune responses [10]. Immunobiotics interact with lymphatic cells via various MAMPs present on their surface, including polysaccharides and LTA; therefore they are recognized by the conserved PRRs of the host [11]. The interplay between gut microbiota and the host drives induction of the tolerance, protection from pathogens, intestinal homeostasis, and induces cytokine production by dendritic cells and macrophages [8]. The motivation for the current study was the need to increase our knowledge of the interaction between bioactive components of the GIT and the host organism. In this simplified model, bioactive components were represented by prebiotics, microorganisms, and synthetic TLR ligands, while the host organism was defined by an ex vivo model of peripheral blood mononuclear cells (PBMC).

The goal of this study was to characterize the cytokine gene expression in the chicken PBMC stimulated with potential immunobiotics. We have tested those immunobiotics in contrast to an array of well-known TLR ligands as reference stimuli. We hypothesize that the chicken PBMC activates cytokine-mediated innate immune responses, which allow for pinpointing molecular mechanisms of immune modulation.

## 2. Materials and Methods

### 2.1. PBMC Culture and Stimulation

PBMC were isolated from the whole blood drawn from seven adult roosters of Green-legged Partridgelike (GP). Whole blood was collected from the wing vein to a tube containing heparin as an anticoagulant. The protocols were approved by the Local Ethics Committee for Animal Experiments (Bydgoszcz, Poland) (study approval reference number 16/2014). Heparinized blood was diluted with HBSS (without Ca^2+^ and Mg^2+^) 1:1 ratio. A volume of 20 mL of diluted blood was transferred to the falcon tube containing 20 mL of 1.077 Histopaque. The falcon tube was centrifuged (centrifuge 5810R, Eppendorf, Hamburg, Germany) at 260× *g* for 30 min at room temperature (24 °C). The PBMC layer was drawn and transferred to a new Falcon tube. Fresh HBSS (without Ca^2+^ and Mg^2+^) was added up to the volume of 30 mL. The falcon tube was again centrifuged at 425× *g* for 20 min. The supernatant was removed, and the cells were rinsed with 20 mL of the fresh HBSS (without Ca^2+^ and Mg^2+^). The suspended PBMC were centrifuged again at 425× *g* for 5 min. The supernatant was removed and the cells were resuspended in 30 mL of RPMI 1640 medium (Biological Industries, Beit Haemek, Israel), supplemented with 10% FBS (Biological Industries, Beit Haemek, Israel), 1% GlutaMAX (Gibco, Dublin, Ireland), and 1% Antibiotic-Antimycotic solution (Gibco, Dublin, Ireland). PBMC were pre-incubated in a culture flask on a shaker for 3 h in a humidified incubator at the temperature of 41.5 °C and atmosphere enriched in 5% CO_2_. After pre-incubation, PBMC were scraped from the flask and transferred to the falcon tube. Cells were centrifuged at 425× *g* for 5 min and resuspended in fresh RPMI 1640 media with supplements as described above (but without 1% Antibiotic-Antimicotic solution). Countess Automated Cell Counter (Invitrogen, Carlsbad, CA, USA) was used to count cells. Viability was estimated using differential cell staining with 0.4% trypan blue (Invitrogen). PBMC were diluted to the working concentration of 1.25 × 10^7^/mL and distributed on 6-well plates (2 mL/well). Cells were incubated overnight prior to stimulation at 42.5 °C in 5% CO_2_. On the day of stimulation, a volume of 100 µL of the respective stimulus was added to the well and mixed by gentle pipetting. The control wells were mock-stimulated with the media. The list of the stimuli is shown in Table 1. PBMC stimulation was conducted in a time-course manner; the cells were harvested at 3 h, 6 h, and 9 h post-stimulation. The experiment was repeated three times (biological replicates) at one-week intervals. 

### 2.2. RNA Isolation and RT-qPCR

PBMC were harvested from individual wells in each experiment (biological replicate). Total RNA was isolated from PBMC cultures using a Universal RNA Purification Kit (EURx, Gdansk, Poland). The concentration and purity of RNA isolates were measured with NanoDrop 2000 spectrophotometer (Scientific Nanodrop Products, Wilmington, NC, USA). rRNA bands integrity were analyzed in 2% agarose gel stained with Simply Safe (EURx, Gdansk, Poland). Cytokine gene expression was determined at mRNA level using the two-step reverse transcription-quantitative PCR (RT-qPCR) method. The amount of 2.5 µg of total RNA was reversely transcribed using Maxima First Strand cDNA Synthesis Kit for RT-qPCR (Thermo Scientific/Fermentas, Vilnius, Lithuania). RT-qPCR gene expression was determined for ten target cytokine genes and two reference genes, based on the oligonucleotide primers reported in Table 2.

### 2.3. Quantitative Reverse Transcription PCR (RT-qPCR)

Complementary DNA (cDNA) was synthesized by using the Maxima First Strand cDNA Synthesis Kit for RT-qPCR (Thermo Scientific/Fermentas, Vilnius, Lithuania), following the manufacturer’s recommendations. Obtained cDNA was diluted to 70 ng/μL working solutions and stored at −20 °C. Each RT-qPCR reaction was conducted in two technical replicates. Cytokine gene panel included the following genes: *IL-1β IL-2, IL-3, IL-4*, *IL-6*, *IL-8*, *IL-10, IL-12p40*, and *IFN-ɣ*. Reference genes used to normalize the samples were *ACTB* and *G6PDH*. The oligonucleotide sequences of the primers are presented in Table 2. RT-qPCR reactions were conducted with a total volume of 10 μL. The reaction mixture included Maxima SYBR Green qPCR Master Mix (Thermo Scientific/Fermentas, Vilnius, Lithuania), 1 μM of each primer (Sigma-Aldrich, Schnelldorf, Germany), and 2 μL of diluted cDNA (70 ng/µl). Thermal cycling was performed in a LightCycler II 480 (Roche Diagnostics, Basel, Switzerland). The thermal program included a step of initial denaturation (15 min at 95 °C), followed by 40 cycles of denaturation (10 s at 95 °C), annealing (15 s at 58 °C), and extension (30 s at 72 °C). Fluorescence was measured at the end of each extension step. After completing the thermal program, the melting curve was generated, which indicated amplification specificity. The thermal program for the melting curve included a gradual increase in the temperature up to 98 °C and measuring the fluorescence of the melting amplicon.

### 2.4. Relative Quantification of Gene Expression and Statistical Analysis

The normalization of the expression levels (Ct—cycle threshold) of the target genes was performed with a geometric mean of the two reference genes (*ACTB* and *G6PDH*). ∆Ct was calculated by subtracting the Ct of the reference genes from the Ct of the target genes (Ct target—Ct reference). All statistical analyses were based on ∆Ct values. One-way analysis of variance (ANOVA) was performed for each time-point independently, using stimuli as the independent variable. The factor was considered significant at *p* < 0.05, *p* < 0.01, or *p* < 0.001. ANOVA was calculated in SAS Enterprise Guide 9.4 (SAS Institute, Cary, NC, USA). A hierarchical cluster tree was constructed in the Multiexperiment Viewer (MeV) version 4.9 [20]. The relative gene expression was calculated with the ∆∆Ct algorithm [21]. The fold change (FC) of the target gene in the experimental group vs. the control group was calculated according to the formula: 2^−∆∆Ct^ [22]. The calculations were performed in MS Excel. and graphs were drawn by using Graph Pad Prism 7 (GraphPad, La Jolla, CA, USA).

## 3. Results

### 3.1. Variance Analysis (One-Way ANOVA)

Supplementary File S1 presents ∆Ct values used for statistical evaluation. Table 3 presents results of variance analysis in which the significance of the stimuli (i.e., TLR ligands or live probiotics) was tested on the cytokine gene expression in the PBMC cultures. The gene expression, measured as dCt values, differed significantly between treatments in *IL1-β*, *IL-6*, *IL-8*, *IL-10*, and *IL-12p40* (*p* < 0.001).

### 3.2. Hierarchical Clustering

**Figure 1:**
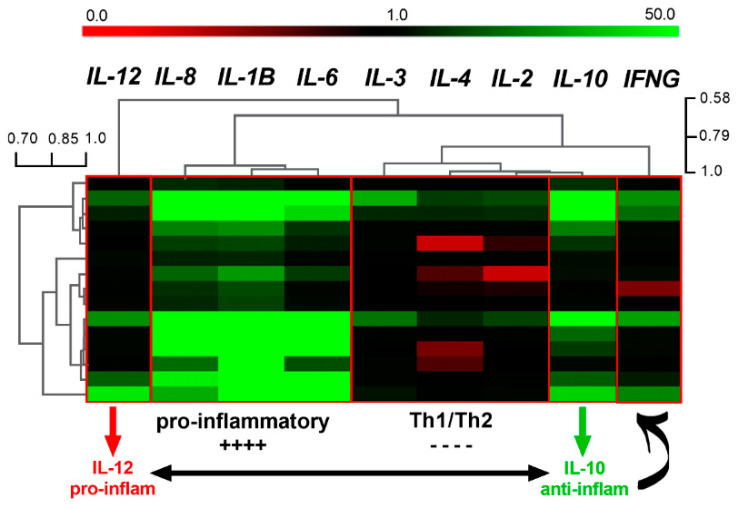
Hierarchical cluster tree of cytokine gene expression data generated from chicken peripheral blood mononuclear cells (PBMC) stimulated with TLR agonists. The cytokine gene panel included: *IL-1*, *IL-2*, *IL-3*, *IL-4*, *IL-6*, *IL-8*, *IL-10*, *IL-12p40*, and *IFN-ɣ* genes. Gene expression analysis performed using RT-qPCR with *ACTB* and *G6PDH* reference genes. Hierarchical Cluster Tree constructed in the Multiexperiment Viewer (MeV) version 4.9.

The gene expression data were clustered into five clusters based on correlation distance (Figure 1). The two largest clusters included: (1) Pro-inflammatory genes (*IL-1β*, *IL-6*, and *IL-8*), which were up-regulated (bright green bars), and (2) Th1/Th2 cytokine genes (*IL-2*, *IL-3*, and *IL-4*), which were down-regulated or unchanged relative to the control (black or red bars). With a large correlation distance from each other were (3) Th1-regulating cytokine genes: *IL-12p40* pro-inflammatory gene and IL-10 anti-inflammatory gene, with *IL-10* being up-regulated (bright green) vs. *IL-12p40* moderately up-regulated (dark green) or unchanged relative to the control (black bars). The gene *IFN-ɣ* was an outlier, located in the node opposite to *IL-12p40*.

### 3.3. Cytokine Gene Expression Analysis

#### 3.3.1. Pro-Inflammatory Cytokines (*IL-1β*, *IL-6*, and *IL-8*)

**Figure 2:**
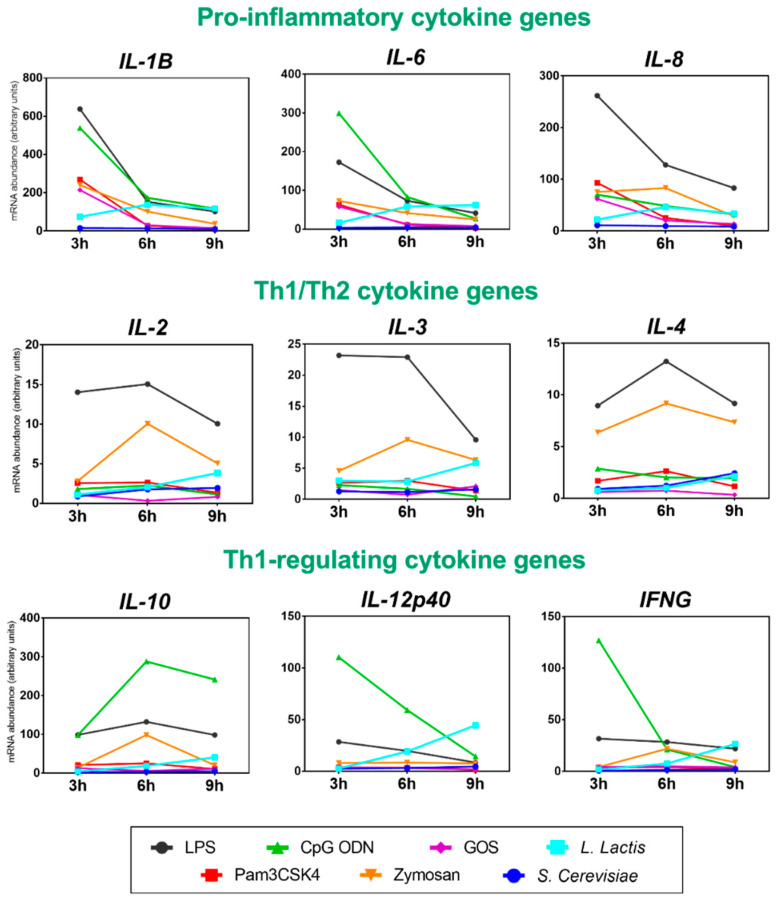
Cytokine gene expression analysis generated from chicken peripheral blood mononuclear cells (PBMC) stimulated with TLR agonists. The PBMC stimulation experiment was performed as follows: 1.25 × 10^7^ cells/well were stimulated with: LPS, CpG ODN, Pam3CSK4, Zymosan, GOS, *Lactococcus lactis* subsp. *cremoris*, and *Saccharomyces cerevisiae*. The stimulation experiment was conducted in a time-course manner and lasted: 3 h, 6 h, and 9 h. Cells were harvested after each time-point for RNA isolation and subsequent RT-qPCR analysis. The cytokine gene panel included: *IL-1*, *IL-2*, *IL-3*, *IL-4*, *IL-6*, *IL-8*, *IL-10*, *IL-12p40*, and *IFN-ɣ* genes. Gene expression analysis performed using RT-qPCR with *ACTB* and *G6PDH* reference genes.

The cytokine gene expression analysis in the chicken PBMC stimulated with various TLR agonists and live probiotics in three different time-points post-stimulation is shown in Figure 2. The first three genes show the pro-inflammatory cluster (*IL-1β*, *IL-6*, and *IL-8*). These cytokines were the most up-regulated, and early responders (3 h), especially to LPS (black line), CpG ODN (green line), Pam3CSK4 (red line), GOS (purple line), and zymosan (orange line). Interestingly, the pro-inflammatory immune response to live probiotics, *L. lactis* (sky blue line) and *S. cerevisiae* (navy blue line) were quite different. Pro-inflammatory immune response to *L. lactis* expressed by mRNA abundance of *IL-1β*, *IL-6*, and *IL-8* genes grew in time, peaked at 6 h, and remained at a high level at 9 h post-stimulation. On the other hand, chicken PBMC did not respond to stimulation with *S. cerevisiae* by activating the pro-inflammatory pathway.

#### 3.3.2. Th1/Th2 Cytokines (*IL-2*, *IL-3*, and *IL-4*)

The second cluster included Th1 (*IL-2* and *IL-3*) and Th2 (*IL-4*) cytokine genes. They were up-regulated with much lower potency compared to pro-inflammatory genes. They were also slower responders as they peaked at 6 h. The Th1/Th2 cytokine gene expression was triggered by LPS and zymosan. Live probiotics (*L. lactis* and *S. cerevisiae*) triggered lower mRNA abundance and towards later time-point of stimulation (9 h).

#### 3.3.3. Th1-Regulators (*IL-10*, *IL12p40*, and *IFN-ɣ*)

Pro-inflammatory *IL-12p40* and anti-inflammatory *IL-10* were clustered on two opposite sides of the cluster tree. It is because those two cytokines have the opposite function. It is quite well represented by mRNA abundance of *IL-10* and *IL-12p40* stimulated by CpG ODN, which initially (3 h) triggered the high gene expression of pro-inflammatory *IL-12p40* together with anti-inflammatory *IL-10*, both at a similar level of mRNA abundance. However, at the 6 h time-point, the relative gene expression of *IL-10* already surpassed *IL-12p40*, leading to its complete silencing at the 9 h time-point. The same pattern of gene expression was expressed by *IFN-ɣ* stimulated with CpG ODN. Live probiotics stimulated different patterns of pro- and anti-inflammatory gene activation. *L. lactis* activated *IL-12p40*, *IFN-ɣ*, and (to a lesser extent) *IL-10* at the 9 h time-point, but *S. cerevisiae* seemed inefficient in triggering the expression of those genes.

## 4. Discussion

In this paper, we determined the kinetics of the cytokine gene expression in the PBMC cells stimulated with (1) well-known TLR ligands, which often serve as vaccine adjuvants (e.g., Pam3CSK4 or CpG ODN), and (2) potential immunobiotics, relatively uncharacterized bioactive compounds (oligosaccharides, prokaryotic and eukaryotic probiotics) used in poultry nutrition and/or veterinary applications. We focused on the first nine hours post-stimulation, which allowed us to pinpoint primary response genes (3 h) and secondary response genes (6 h and 9 h). This way, we characterized the overall cytokine gene expression triggered in PBMC by the respective stimuli.

### 4.1. Hierarchical Clustering

The gene expression data were first clustered using correlation distance. The most pronounced cluster included strongly up-regulated pro-inflammatory cytokines (*IL-1β*, *IL-6*, and *IL-8*). Pro-inflammatory cytokines orchestrate inflammation, which is a complex response of the immune system to the loss of homeostasis due to tissue stress, injury, and infection [23]. The inflammatory response is controlled by transcriptional activation of the gene sequences by three classes of transcriptional factors, activating primary response genes (Class I transcription factors), secondary response genes (Class II transcription factors), and macrophage-specific gene expression (Class III transcription factors) [24]. Typically, Class I transcription factors, including NF-ĸB and IRF, are activated post-translationally by the receptors of the innate immune system [24]. For example, the acute inflammatory responses are triggered by the microbial components (e.g., LPS) recognized by the respective TLR (e.g., TLR4). Acute inflammatory responses have also been regulated by other molecular mechanisms. Shen, et al. [21] demonstrated that the LPS-activated pro-inflammatory cascade in PBMC isolated from broiler chickens had epigenetic character. In particular, LPS stimulation triggered demethylation of some CpG islands within promoters of *IL-6* and *TNF-α* genes, as well as increased availability of *IL-1β* gene promoter due to decreased chromatin compactness.

The second large cluster of the genes was classified as Th1/Th2 cytokine genes and was correlated based on a much lower gene expression pattern compared to pro-inflammatory genes discussed above. The second gene cluster included three cytokine genes: *IL-2*, *IL-3*, and *IL-4*. Those cytokine genes participate in the immunological decision-making process [25]. In particular, the Th1 and Th2 cytokines skew naïve T cells into mounting either Th1 or Th2 immune responses. Th1 cells secrete *IL-2* and Th2 cells secrete *IL-3* and *IL-4* cytokines. Polarization of the naïve T cells into any of the Th phenotypes is associated with the adaptive (specific) immune responses attributed to the affinity of the T-cell receptor (TCR) to the antigen. Activation of the naïve T cells requires the presence of the particular antigen-presenting cells, which are called Dendritic Cells (DC). Upon infection, DC recognizes PAMPs with the conserved array of various TLRs present on their surface. DC engulfs the antigens and presents their digested fragment via the MHC class II region to the TCR. Together with the additional signals required for the first activation of the naïve T cells, primed DC can activate T cells into the development of the adaptive immune responses [26]. This way, DC bridge innate and adaptive immune responses.

Th1/Th2 cytokine gene expression can be detected in PBMC sourced from different organisms, including chicken [27], pig [28], and cattle [26]. T cell activation depends to a large extent on the stimuli used, time-point post-stimulation analyzed, and the immune competence of the organism that sourced the blood for PBMC. Kirthika et al. [28] compared cytokine gene expression in PBMC sourced from two distinct swine genotypes stimulated in vitro with phytohemagglutinin (PHA). PHA is a mitogen that stimulates the proliferation of the T cells. PBMC sourced from indigenous, well-adapted pig breed from India responded in much higher Th1 and Th2 gene expression signatures compared to PBMC sourced from a conventional commercial pig breed. Porcine PMBC stimulated with PHA expressed the highest abundance of *IL-2* mRNA at 24 h post-stimulation and *IL-4* at 2 h post-stimulation [29]. In the current study, a Th1/Th2 cluster expressed low mRNA abundance, but the only mitogen used was LPS, which stimulates the proliferation of B cells but not the T cells. PBMC analyzed in this study were sourced from GP chickens, which belong to a native, dual-purpose breed, known for its resilience and distinct immune responses [30,31].

The remaining genes were clustered as follows: IL-10 was clustered on the side of Th1/Th2 cluster and the remaining cytokines (*IL-12p40* and *IFN-ɣ*) were outliers on the heat map. However, due to the functional relationship, those genes need to be analyzed together. We called the last cluster Th1-regulating cytokine genes because *IL-12p40* skews lymphocytes T towards Th1 immune responses, whereas *IL-10* negatively regulates Th1 activation [32]. As such, *IL-10* also down-regulates *INF-ɣ* cytokine gene expression, which is secreted by Th1 cells [33]. If we consider inflammatory processes described earlier, there are both physiological and pathological consequences to inflammatory immune responses. Physiologically, inflammation aims to protect the host organism against the microbial burden and restore homeostasis. On the other hand, the excessive or uncontrolled inflammatory responses promote tissue damage and autoimmune responses, which unbalance the homeostasis [23]. For this reason, the anti-inflammatory *IL-10* cytokine gene expression was activated, which counter-balances pro-inflammatory cytokine *IL-12p40*, and plays a protective role in the PBMC population against excessive inflammation.

### 4.2. Gene Expression Patterns

#### 4.2.1. Pro-Inflammatory Cytokines (*IL-1β*, *IL-6*, and *IL-8*)

More detailed analysis of the gene expression patterns activated in chicken PBMC in vitro shows that the pro-inflammatory cytokine storm was activated by two major PAMPs: LPS (*IL-1β*, *IL-6*, and *IL-8*) and CpG ODN (*IL-1β*, *IL-6*, *IL-12p40*, and *IFN-ɣ*). In both cases, the acute inflammatory responses were most pronounced at 3 h post-stimulation, with a gradual decrease at 6 h and 9 h post-stimulation. Even though both PAMPs stimulated the mRNA abundance of the effector cytokines similarly, the underlying molecular mechanism is quite distinct. LPS is a major constituent of the outer membrane of all Gram-negative bacteria. LPS is also a biologically active endotoxin, which stimulates immune responses in a dose-dependent manner. It contains two components: lipid A, associated with the toxic effect, and a polysaccharide, eliciting inflammatory immune responses [34]. LPS binds TLR4 and activates the NF-ĸB transcription factor, which stimulates downstream production of pro-inflammatory cytokines and B cell proliferation [35]. LPS is one of the most potent immunomodulators. In small doses, it triggers acute inflammatory responses in vitro and in vivo, but in large doses, it is lethal [36]. As LPS is the model stimuli, it’s application in research can be considered as a reference point for less characterized bioactive compounds. For example, LPS induced a fold change of 637 in *IL-1β* mRNA abundance at 3 h post-stimulation. Zymosan was comparable with GOS (>200 fold change at 3 h), but *L. lactis* peaked at 6 h with ~140 fold change and *S. cerevisiae* induced much lower up-regulation of *IL-1β* mRNA (~15 at 3 h and 6 h).

#### 4.2.2. Th1/Th2 Cytokines (*IL-2*, *IL-3*, and *IL-4*)

Th1/Th2 polarization cytokines have been activated on a much lower level compared to pro-inflammatory cytokines. The peaks of *IL-2*, *IL-3*, and *IL-4* mRNA abundance were at 6 h post-stimulation and the two stimuli that activated those cytokine genes were LPS and zymosan. *IL-2* is a cytokine that is produced during the primary immune response by naïve Th cells. Upon differentiation of Th cells into Th1 or Th2 phenotypes, they reduce *IL-2* secretion and take up production of the respective Th1-type (*IFN-ɣ*) or Th2-type (*IL-4*) cytokines [37]. In contrast, *IL-3* is a T cell-derived multilineage hematopoietic growth factor, that is required for survival, proliferation, and differentiation of the primitive hematopoietic progenitor cells [38]. Zymosan is a crude component of the yeast’s cell wall, containing glucans (55%), mannans (19%), and chitins [39]. It elicits unique immunomodulatory effects in vitro, associated with the glucan fraction.

#### 4.2.3. Th1-Regulators (*IL-10*, *IL12p40*, and *IFN-ɣ*)

CpG ODN was the stimuli that activated the most potent response of the pro-inflammatory (Th1-type) and the anti-inflammatory (Th2-type) cytokines (Figure 2). CpG ODN refers to bacterial oligonucleotides rich in unmethylated CpG dinucleotides. In mammals, CpG ODN is an agonist of TLR9, which in avian species is represented by a functional orthologue, TLR21 [40]. Unlike other TLRs, which are expressed on the cell surface, TLR21 is an endosomal receptor. It recognizes a broad range of CpG ODN motifs from internalized bacteria and triggers NF-ĸB-mediated expression of pro-inflammatory (Th1-type) cytokines, including *IL-1β*, *IL-6*, *IL-12p40*, and *IFN-ɣ* as well as anti-inflammatory (Th2-type) cytokine *IL-10*. The broad spectrum of immune activation by different sequences of CpG ODN made them promising candidates for versatile vaccine adjuvants, used to boost the effectiveness of protein-based vaccines against allergies, infectious diseases, and cancer [41]. In poultry, immunostimulatory CpG ODN was applied orally to day-old chickens and followed by *Salmonella enteritidis* challenge, reduced pathogen invasion, and chicken mortality [42].

In this study, CpG ODN induced potent activation of Th1-type stimulating cytokine genes, *IL-12p40* and *IFN-ɣ* at 3 h post-stimulation, followed by their rapid down-regulation at 6 h and 9 h. Reversely, mRNA abundance of *IL-10* cytokine gene peaked at 6 h post-stimulation and remained high at 9 h post-stimulation. The *IL-12p40* and *IL-10* cytokines indicate the subtle balance between the stimulatory (pro-inflammatory) and regulatory (anti-inflammatory) effect of the given immunobiotic on the antigen-presenting cells (APC), such as macrophages, DC, and B cells [32]. Upon antigen recognition, engulfing, and presentation via MHC class II, the respective APC secretes cytokines that direct Th development toward Th1-type or Th2-type immune responses. CpG ODN induces B cell activation and prime macrophages to secrete *IL-12* cytokine. Indirectly, *IL-12* cytokine secreted by activated macrophages acts on natural killer (NK) cells to secrete *IFN-ɣ* [43]. The pleiotropic *IL-10* cytokine produced by primed macrophages and DC cells (but not B cells) inhibits stimulation of *IFN-ɣ* production by Th1 lymphocytes [44]. As such, *IL-10* serves as a feedback regulator and protects from excessive pro-inflammatory responses mediated by the primed APC.

In contrast, zymosan showed distinct stimulatory properties towards cytokines in the group of Th-1 regulators. Stimulation with zymosan increased *IL-10* and *IFN-ɣ* mRNA abundance, but only at the 6 h time-point, while *IL-12p40* mRNA abundance remained unchanged. A different pattern of gene expression regulation triggered by zymosan can be explained by its molecular recognition. It is known that zymosan activates macrophages and DC via TLR2 and Dectin-1 [45]. In an elegant study on human DC, Dillon et al. [46] demonstrated that zymosan promoted immune tolerance by inducing DC to secrete regulatory *IL-10* and suppressing pro-inflammatory *IL-12*. As such, zymosan is now considered a suppressive molecule that increases immune tolerance via maintaining high levels of *IL-10* cytokine during infection. Due to this property, zymosan is a promising target for autoimmune, allergy, and transplantation therapies.

### 4.3. Cytokine Gene Stimulated by Immunobiotics

Based on presented cytokine gene expression patterns in chicken PBMC, we characterized the immunomodulatory role of GOS prebiotic and two live probiotics, i.e., *L. lactis* and *S. cerevisiae*. Each of those compounds stimulated cytokine gene expression in the chicken PBMC in a different manner. GOS induced the strongest up-regulation of the pro-inflammatory cytokine genes, especially *IL-1β* (~214 fold change), *IL-8* (~62 fold change), and *IL-6* (58 fold change) at 3 h post-stimulation. Oligosaccharides have been known to trigger immune responses both in vitro and in vivo. Vendrig, et al. [47] determined that GOS dose-dependently enhanced the pro-inflammatory immune responses of equine PBMC upon LPS challenge. Quite reversely, human PBMC cultured with GOS and challenged with LPS secreted a lower amount of immune mediators (e.g., *IL-1α* and *IL-1β*), compared to LPS challenge alone [48]. The primary stimulant of GOS has been associated with the low molecular weight molecules, such as tri- and tetrasaccharide fractions [49].

*L. lactis* triggered the strongest up-regulation of the pro-inflammatory cytokine genes (*IL-1β*, *IL-6*, and *IL-8*) at 6 h post-stimulation and Th1-regulators (*IL-12p40*, *IL-10*, and *IFN-ɣ*) at 9 h post-stimulation. Foligne et al. [50] proposed a screening test using PBMC to select the probiotic strains that promote the release of anti-inflammatory cytokines. These probiotics were ranked based on the IL-10/IL-12 cytokine ratio, indicating their ability to attenuate inflammatory bowel disease in vivo. In this ranking, *L. lactis* performed rather poorly, with an IL-10/IL-12 cytokine ratio around 1 [50]. In the current study, the tested strain of *L. lactis* activated a higher abundance of mRNA expression of the *IL-12p40* pro-inflammatory cytokine gene rather than *the IL-10* anti-inflammatory gene, which classifies it as an immunostimulatory probiotic. On the other hand, *S. cerevisiae* analyzed in this study did not activate immune-related gene expression signatures in chicken PBMC. *S. cerevisiae* (yeast) has been successfully used as probiotics for poultry for years [51]. They have also been known for their potential to activate the immune system of the animal [52] or bind pathogenic enterobacteria [53]. The immunomodulatory components of the yeast are cell wall polysaccharides, such as mannan-oligosaccharides and β-glucan, or MAMP, such as zymosan. Other bioactive compounds that can stimulate PBMC are fermentation products such as XPC [54]. In this study, we used purified yeast culture for PBMC stimulation, which was not an efficient mode of PBMC stimulation, most likely due to the low availability of the TLR ligands.

In comparison to the purified TLR agonists, the immune responses elicited by the live *L. lactis* bacteria were shifted in time. LPS or CpG ODN triggered the strongest gene expression up-regulation of the pro-inflammatory cytokines or Th1-regulators at 3 h post-stimulation, whereas, *L. lactis* activated the respective genes at 6 h and 9 h post-stimulation. The beneficial effects of *L. lactis* and other immunobiotics have been widely attributed to the activity of exopolysaccharides (EPS) [55]. EPS are polysaccharides that are either loosely bound to the cell wall in a capsular form or secreted to the environment [56]. Among Gram-positive bacteria, there are several EPS-producers, including different strains of *L. lactis* subsp. *cremoris* [57,58]. Many of them released EPS that conferred specific immunomodulatory effects in the host, such as induction of *IL-1α* and *IFN-ɣ* in spleen macrophages [59], induction of macrophage cytotoxicity [60], and mitogen activity in lymphocytes [61]. Hereby, we hypothesize that the cytokine gene expression activated by *L. lactis* was mediated indirectly by EPS rather than the by direct contact with bacterial PAMPs.

## 5. Conclusions

In conclusion, we have determined cytokine gene expression in chicken PBMC stimulated with an array of purified TLR ligands and potential immunobiotics. We found pro-inflammatory Th1/Th2 and Th1-regulator gene clusters. Among the TLR ligands, the strongest stimuli of PBMC were LPS and CpG ODN. Among bioactive compounds, *L. lactis* subsp. *cremoris* triggered more abundant cytokine gene expression, but *S. cerevisiae* seemed inefficient in triggering immune-related gene expression. Immune responses of PBMC to *L. lactis* had a pro-inflammatory character and was expressed by increased mRNA abundance of *IL-1β*, *IL-6*, *IL-8*, and *IL-12p40*. In this manner, *L. lactis* subsp. *cremoris* has immunostimulatory properties in chicken PBMC and therefore can be considered an immunobiotic.

## Figures and Tables

**Table 1:** Toll-like receptor (TLR) ligands and live probiotics are used to stimulate chicken peripheral blood mononuclear cells (PBMC) in vitro.

TLR Ligand	Description	chTLR	Dose	Source
LPS	Lipopolysaccharide from *E. Coli* 026:B6	TLR4	2.5 µg/mL	Sigma-Aldrich (L2654)
CpG ODN	Short, synthetic, single-stranded DNA molecules, containing unmethylated CpG motifs; these CpG motifs are present at 20-fold greater frequency in bacterial DNA compared to mammalian DNA	TLR21	10 µg/mL	Invivogen (tlrl-m362)
Pam3CSK4	Synthetic diacylated lipopeptide (LP); Bacterial lipopoliproteins are strong activators of innate immune responses, present in cell walls of Gram (+) bacteria	TLR2	10 ng/mL	Invivogen (tlrl-pm2s-1)
Zymosan	Cell wall preparation of *S. cerevisiae*	TLR2 and TLR6	10 µg/mL	Invivogen (tlrl-zyn)
GOS	Galactooligosaccharides, oligosaccharides composed of galactose units, produced from milk lactose	Uncharacterized	100 µg/mL	In-house
*L. lactis* subsp. *cremoris*	Lactic Acid Bacteria (LAB), Gram-positive, belong to *Eubacteriales* order, *Streptococcaceae* family, used as probiotic and in diary products	Putative TLR2	1.25 × 10^8^/mL	In-house
*S. cerevisiae*	Eucaryotic, single-cell fungus (yeast), antagonistic to pathogenic bacteria, known probiotic in many medical applications	Putative TLR2 and TLR6	1.25 × 10^8^/mL	In-house

**Table 2:** Oligonucleotide primer sequences for RT-qPCR.

Gene	NCBI ID	Primer (5’->3’)	References
*IL-1β*	395196	F: GGAGGTTTTTGAGCCCGTC R: TCGAAGATGTCGAAGGACTG	[12]
*IL-2*	373958	F: GCTTATGGAGCATCTCTATCATCA R: GGTGCACTCCTGGGTCTC	[13]
*IL-3*	474356	F: GCAGCAATGAAGCCATACCT R: GTGACTGCATTCTCTTCCCCT	This study ^1^
*IL-4*	416330	F: GCTCTCAGTGCCGCTGATG R: GGAAACCTCTCCCTGGATGTC	[13]
*IL-6*	395337	F: AGGACGAGATGTGCAAGAAGTTC R: TTGGGCAGGTTGAGGTTGTT	[14]
*IL-8*	396495	F: AAGGATGGAAGAGAGGTGTGCTT R: GCTGAGCCTTGGCCATAAGT	[15]
*IL-10*	428264	F: CATGCTGCTGGGCCTGAA R: CGTCTCCTTGATCTGCTTGATG	[16]
*IL-12p40*	404671	F: TTGCCGAAGAGCACCAGCCG R: CGGTGTGCTCCAGGTCTTGGG	[17]
*IFN-* *ɣ*	396054	F: ACACTGACAAGTCAAAGCCGC R: AGTCGTTCATCGGGAGCTTG	[17]
*ACTB*	396526	F: CACAGATCATGTTTGAGACCTT R: CATCACAATACCAGTGGTACG	[18]
*G6PDH*	AI981686	F: CGGGAACCAAATGCACTTCGT R: GGCTGCCGTAGAGGTATGGGA	[19]

^1^ Primers were designed using PrimerBLAST (https://www.ncbi.nlm.nih.gov/tools/primer-blast).

**Table 3:** Results of variance analysis (one-way ANOVA) at different time-points post-stimulation.

Gene	3 h	6 h	9 h
*IL-1β*	<0.001	<0.001	<0.001
*IL-2*	ns	<0.05	ns
*IL-3*	ns	ns	ns
*IL-4*	ns	ns	ns
*IL-6*	<0.001	<0.001	<0.001
*IL-8*	<0.001	<0.05	<0.05
*IL-10*	<0.001	<0.001	<0.001
*IL-12p40*	<0.001	<0.001	<0.001
*IFN* *-* *ɣ*	ns	ns	ns

*p*-value, levels of significance: <0.05, <0.01, <0.001, ns (not significant) >0.05.

## Data Availability

The data presented in this study are available in Supplementary File S1.

## Supplementary Materials

The following are available online at https://www.mdpi.com/2073-4425/12/2/195/s1, Supplementary file S1, Delta-Ct values from RT-qPCR used for statistical evaluation.
